# Editorial: Endocrine consequences of sleep disorders

**DOI:** 10.3389/fendo.2023.1238950

**Published:** 2023-06-29

**Authors:** Karin Harumi Uchima Koecklin, Tatsuo Shimosawa, Peng Li

**Affiliations:** ^1^ Life Sciences Institute, University of Michigan, Ann Arbor, MI, United States; ^2^ Department of Biologic and Materials Sciences, School of Dentistry, University of Michigan, Ann Arbor, MI, United States; ^3^ Department of Molecular and Integrative Physiology, School of Medicine, University of Michigan, Ann Arbor, MI, United States; ^4^ Department of Clinical Laboratory, Faculty of Medicine, International University of Health and Welfare, Chiba, Japan

**Keywords:** sleep disorders, endocrine disorders, obstructive sleep apnea, obesity, thyroid dysfunction, sleep apnea, circadian disruption, diabetes

Sleep is a vital physiological process necessary for sustaining both physical health and optimal brain function (1–3). Despite the recommended sleep duration of 7 to 9 hours for adults, achieving this has become increasingly challenging in today’s society due to the rising rates of sleep disorders and sleep deprivation (4, 5). Disruption in sleep has been associated with various endocrine disorders, such as obesity, type 2 diabetes mellitus, and thyroid hormone dysregulation. This Research Topic strives to enhance our comprehension of the intricate relationship between sleep and endocrine disorders, with the ultimate goal of advancing the treatment approaches for these pathological conditions.

One of the most prevalent sleep disturbances is sleep apnea, specifically obstructive sleep apnea (OSA), which affects nearly 1 billion adults worldwide between the ages of 30 and 69 (6, 7). OSA is closely associated with obesity, as 40% of people with a BMI over 30 also have OSA (7). Obesity is considered a significant risk factor for the development and progression of OSA, while OSA also contributes to weight gain and difficulty in losing weight. In this Research Topic, the study of Ma et al. focused on the association between OSA and abdominal fat distribution in adult Chinese patients with obesity. This cross-sectional study found that the prevalence of OSA in obese patients was high, with increased neck circumference in patients with severe OSA, and increased visceral fat area in all patients with OSA. Moreover, the abdominal visceral adipose tissue accumulation was also increased in obese patients with OSA and posed as a risk for the development of OSA. These findings have implications for the diagnosis and treatment of OSA in obese patients, highlighting the importance of considering these factors in clinical approaches.

A common treatment option to promote weight loss in obese patients is bariatric surgery. Approximately 45% of obese patients with OSA undergo this procedure, which not only aids in weight reduction but also improves their sleep quality and OSA condition (8). In line with this, Leentjens et al. investigated on the level of angiopoietin-like protein 7 (ANGPTL7), a member of the ANGPTL family that play a role in inducing insulin resistance, in patients with different severity of OSA who underwent bariatric surgery by conducting a prospective cohort study in Netherlands. ANGPTL7 exhibited a positive correlation with the apnea-hypopnea index in moderate and severe OSA cases. Interestingly, bariatric surgery significantly decreased the levels of ANGPTL7 in all patients, regardless of the severity of OSA, accompanied by a decrease in the apnea-hypopnea index. These findings highlight the potential involvement of ANGPTL7 in OSA and suggest its potential utility as a diagnostic and prognostic marker in OSA patients undergoing bariatric surgery.

Type 2 diabetes mellitus (T2DM) is another pathological condition that is associated with OSA (9). Understanding the association between OSA and T2DM is crucial because both conditions are complex and can have overlapping pathophysiological mechanisms. The study of Worku et al. further addressed the risk of OSA in T2DM patients and its predictors with a cross-sectional study in Ethiopia. Their results showed a nearly 32% prevalence of OSA in T2DM patients. Other risk factors for OSA they reported include male sex, physical inactivity, higher neck circumference, and comorbid hypertension. Similarly, Guo et al. studied the relationship between T2DM and the respiratory event duration in patients referred to a sleep clinic for the diagnosis of OSA. Their results showed that a decreased respiratory duration increased the odds of T2DM while a shorter apnea duration would be not only associated with this disease, but also with poor sleep quality and lower arousal threshold. Therefore, these studies provide valuable insights into the significance of evaluating and assessing OSA in T2DM patients.

To further complicate the relationship between T2DM and sleep disturbance, it is important to consider that not only does the disease itself is associated with sleep issues, but the medical treatment of T2DM can also impact sleep quality (10). T2DM patients usually present sleep difficulties, which could be a side effect of the medicine used to manage hyperglycemia in these patients. Taking this into consideration, Xue et al. evaluated the association between oral antidiabetic therapy and sleep in T2DM patients from the UK Biobank. Through the analysis of a large cohort, the study revealed that patients using non-metformin medications had a higher likelihood of experiencing difficulties in both falling asleep and staying asleep compared to both metformin users and untreated patients. Additionally, non-metformin users presented longer sleep duration than untreated and metformin patients. These findings underscore the significance of considering the choice of antidiabetic medication and its potential association with sleep disturbances. Taken together, these studies suggest that identifying and addressing sleep issues in T2DM patients can help optimize the treatment outcomes and enhance overall quality of life.

Another endocrine problem that is associated with sleep disturbances is thyroid dysfunction. Green et al. discussed in their review about the effects of thyroid dysfunctions on sleep, highlighting the implication of thyroid hormone dysregulation in various sleep disturbances. In light of this, it is important to note that untreated conditions such as hyperthyroidism and hypothyroidism can significantly impair both the quantity and quality of sleep, consequently exerting a profound influence on the overall health of other body systems.

Circadian rhythm has been suggested to be associated with metabolic health and food intake (11, 12). The perspective article of Marino and Arble provided valuable insights into the mechanisms linking peripheral clock disruption and metabolic diseases by reviewing multiple studies. The authors highlight the significance of cyclic clock genes in peripheral tissues and their association with metabolic disorders, which provides insights into the underlying mechanisms that contribute to metabolic disease in relation to circadian disruption and diverse sleep disorders.

In this Research Topic, we present seven studies that explore various facets of the intricate relationship between sleep and endocrine disorders. These findings offer valuable insights that can contribute to a more informed clinical approach toward these interconnected phenomena, particularly as their incidence continues to rise each year.

## Author contributions

All authors have contributed to the preparation of the editorial and have reviewed and approved the final submitted version.
